# Acid Etching's Effect on Hypomineralized Teeth's Bond Strength and Enamel Surface Structure

**DOI:** 10.7759/cureus.66909

**Published:** 2024-08-14

**Authors:** Maha M.M. Ali, Mena F Abdallah Ali, Noura M Bakr, Mennat Allah M Shendy, Wafik A Saleh, Amany M Diab, Ahmed K Algariah

**Affiliations:** 1 Orthodontics, Faculty of Dental Medicine for Girls, AL-Azhar University, Cairo, EGY; 2 Oral Biology, Faculty of Dental Medicine for Girls, AL-Azhar University, Cairo, EGY; 3 Dentistry, Ministry of Health Holdings, Cairo, EGY; 4 Pediatric Dentistry, Sinai University, Faculty of Dentistry, Arish, EGY; 5 Orthodontics, Sinai University, Faculty of Dentistry, Arish, EGY

**Keywords:** enamel, orthodontic brackets, shear bond strength, acid etching, hypomineralized teeth

## Abstract

The current study evaluated the bracket bond strength of hypomineralized teeth with and without acid etching. Twenty premolar teeth extracted for orthodontic purposes were used in this study. Hypomineralization was induced in all experimental teeth using a cariogenic solution. Then the teeth were divided into two groups. In the first group, orthodontic brackets were bonded by orthodontic adhesive (Green Gloo, Ormco, USA) without etching, while in the second group, further etching was used before bonding. All specimens were examined for morphological changes in the enamel surface after demineralization and debonding using a scanning electron microscope. The shear bond strength was also measured, and the recorded values of bond strengths were collected and statistically analyzed. All massed results were statistically evaluated via an independent t-test to equate Group 1 (G1) and Group 2 (G2). A p-value of < 0.05 is deemed to be statistically substantial. The shear bond strength of groups interacted by orthodontic adhesive with etching (Group 2) was considerably greater than that of groups bonded by orthodontic adhesive without etching. The etching indicates considerably greater tackiness rates compared to bonding without etching, although it leaves more destructed cores, which may need further treatment.

## Introduction

Enamel is made up of 87% highly packed hydroxyapatite crystals, which makes it resistant to mechanical abrasion and increases its hardness [1]. The characteristics of the enamel surface determine whether the bonding process for orthodontic brackets will be successful. A disease known as molar hypomineralization or environmental variables like white spot lesions can sometimes cause patients who need orthodontic treatment to have demineralized enamel [2]. The acid etch process is vital for the formation of the perfunctory union between lacquer and complex dental mastics. It contains numerous stages that might impact the union intensity of the complex resin with the enamel [3]. Etching refers to the incomplete breakdown of enamel crystals, which aids mechanical maintenance between the orthodontic tag and tissue holes. Etching provides a substantial irregularity on the incisor exterior, which is important for the braces' attachment stability [4-7 ]. Acid imprint is crucial in odontology since it can provide a prototype for the acid damage initiated by cavities and aid in the taxonomic and evolutionary categories of mammals at the beginning of fundamental changes in enamel [ 8-11]. Furthermore, this procedure is quite vital in odontology treatment since it enables the adhesion of corrective substances to the quarries and cracks it produces [12]. It seems that etching with phosphoric acid is capable of producing thoroughly clean enamel and dentin surfaces [13]. A debate among practitioners regarding whether resin substances are best for treating hypomineralized affected teeth was issued. Bonded repairs have been effective in restoring teeth to adequate structure, purpose, and esthetics in certain individuals with hypomineralized teeth. Bonded restorations, on the other hand, have a significant failure rate due to insufficient adhesion between the restoration and the enamel [9]. These failures were caused by chemical and structural variations between sound and hypomineralized enamel [10,11,14 ]. The enamel and/or dentin composition and structure alter when there are dental structural problems. As a result, bonding glue procedures grow more complex and challenging [15].

The study tries to understand more about hypomineralized teeth that require orthodontic fixed appliances, which is a critical issue for both patients and orthodontists.

This research was performed to assess the impact of etching or not etching on the bond strength of brackets on hypomineralized teeth and the effect on enamel surface structure. The theories examined were that: 1. The bond strength of hypominerlized enamel varies between bonding with etching and without etching. 2. There is a difference in hypomineralized enamel surface between bonding and etching.

## Materials and methods

Ethical approval

The experiment was done according to the recommendations and approval of the Ethics Committee of the Faculty of Dental Medicine for Girls, Al-Azhar University, for working on extracted human teeth (Approval No. P-PD-21-09). Extracted premolars were collected from orthodontic patients after obtaining their consent to have their teeth included in the study. Teeth without deformities or cracks were selected for the study and kept in physiological saline. Specimens were distributed into two groups (n=10 per group).

Sample size calculation

**Figure 1:**
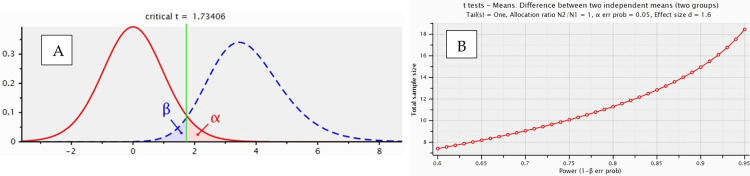
Sample size estimation (sample size group 1=10, sample size group 2=10, overall sample size = 20) A: Graphic representation of effect size, B: Power test analysis

Figure 1: f is the effect size of 1.60, α is 0.05, and Power (1-β) = 0.95. 

Preparation of teeth for hypo mineralization

The buccal surface of the teeth was properly cleaned and then covered by a layer of acid-resistant varnish, leaving an uncovered square area in the center.

All the teeth were immersed for 12 weeks in a cariogenic solution consisting of 2.2 mM CaCl2, 2.2 mM NaH2PO4, and 50 mM acetic acid (PH 4.8) to create demineralized enamel. This solution was replaced weekly. After that, the demineralized teeth were rinsed with tap water [2]. The buccal surfaces of the teeth in the experimental groups were cleaned with a water slurry of pumice and rubber prophylactic cups for 5 seconds and then underwent the following surface preparation procedures. The teeth were divided into two groups of ten teeth each. Group 1: stain steel orthodontic brackets bonded with orthodontic glue (Green Gloo, Ormco, USA) without acid etching before bonding. Group 2: conventionally bonded brackets (Mini 2000, Ormco, USA) with a contact surface area of 20.90 mm2. Each tooth was etched for 30 seconds with orthophosphoric acid (Orthodontic Bonding System, Acid Etch, Ormco, United States), washed under pressure, and dried. After that, a traditional orthodontic compound was used to glue the teeth together (Green Gloo, Ormco, USA) along with its proprietary bond (Solo Bond, Ormco, USA). A standard tungsten quartz halogen curing light (Optilux 501, Demetron Research Corporation) was used for curing. A light intensity range of between 440 and 480 mW/cm2 was used with a distance of 1 mm from the bracket/tooth interface for 40 seconds total and 10 seconds from each side of the bracket.

Electron microscope scanning

At the Desert Research Centre in Cairo, teeth were studied for morphological changes in the enamel surface following demineralization and debonding (48 hours after bonding) using a scanning electron microscope (Philips XL 30).

The buccal surface of the teeth was gold-plated before bonding and after debonding of the brackets to increase the conductivity of the scanning electron microscope. To do so, the teeth were covered by 20 or 30 microns of gold using the Hummer 8 Sputter Coater machine.

Bond strength test

**Figure 2:**
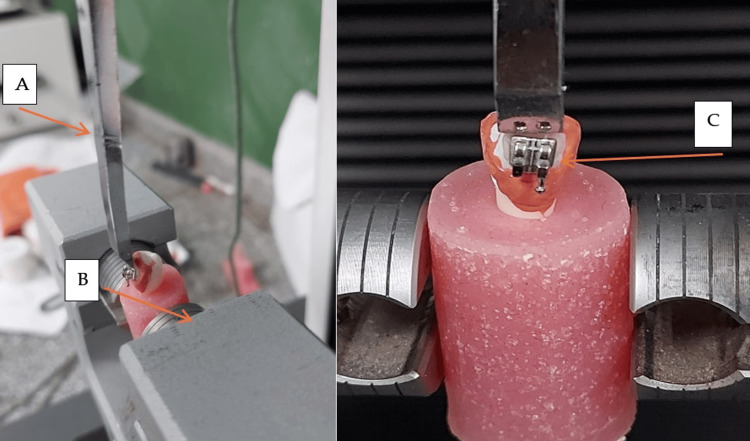
Procedures for measuring bond strength are shown in frontal and lateral perspectives. A: chisel-molded rod. B: an Instron universal testing machine. C: tooth –bracket contact

The buccal surface of each tooth was aligned vertically to the base of the mold using a mounting jig to embed it in acrylic resin. The samples were then placed in a jig that was connected to an Instron universal testing instrument (Model 3345, Instron, UK). The specimens were fastened in the machine's lower jaw, and the bracket base was parallel to the shear force path. A strong chisel-molded rod affixed to the side of the Instron machine was used to apply continuous shear force as close to the tooth/bracket contact as feasible at a crosshead rate of 1 mm per minute until the bracket separated, as illustrated in Figure 2.

Statistical analyses

IBM Corp. Released 2019. IBM SPSS Statistics for Windows, Version 26.0. Armonk, NY: IBM Corp. was used to conduct all statistical analyses. To compare Group 1 and Group 2, all the obtained data was computed, tabulated, and statistically examined using an independent T-test. A statistically significant p-value is less than 0.05.

## Results

Load measurement

Statistical analysis revealed significant variation among parties (p<0.001). The highest maximum load was recorded in group 2 (194.66±0.51) than in group 1 (101.31±0.56). Values recorded by the bond strength test for brackets bonded without etching were lower than those of brackets bonded with etching (Table 1).

**Table 1:** Bond strength measurement *means a significant difference at p<0.05. Table 1 shows that the bond strength of brackets to hypomineralized teeth without etching has lower values than that with etching.

	Mean	SD	95% Certainty Interval of the Difference		Indep.T-test	p-value <0.05
			Lesser	Superior		
Group 1	101.31	0.56	-93.86	-92.85	389.04	<0.001*
Group 2	194.66	0.51	-93.86	-92.85		

**Figure 3:**
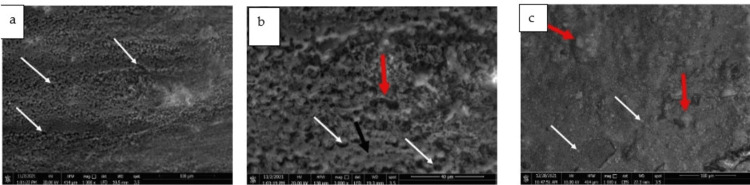
3a: SEM micrographs demonstrating an irregular surface of hypomineralized enamel containing many defects (white arrows). (Magnification X1000), 3b: Higher magnification of Figure 3a showing: type-I etching pattern (white arrows), destruction of the core and part from the wall (red arrow), and pit formation. (Black arrow). (Magnification X3000), 3c: SEM micrograph showing a layer of bonding material covering the surface with granular (white arrows) and globular deposits (red arrows). (Magnification X1000)

**Figure 4:**
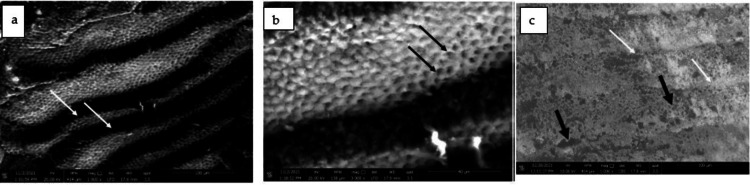
4a: SEM micrographs demonstrating etched enamel with pronounced perikymata (white arrows). (magnificationX1000), 4b: Higher magnification of Figure 4a showing a typical honeycomb pattern of etched enamel (white arrows). (Magnification X3000), 4c: SEM micrograph showing relatively smooth enamel surface with some defective areas (black arrows) and perikymata became shallower (white arrows) (magnification X1000).

SEM examination of the surface of etched (hypomineralized) enamel revealed an irregular surface with randomly distributed small defects, representing a disrupted prism pattern (Figure 3a). On higher magnification, the type I etching pattern was clear, where the central prism core was partially or completely lost, and other types of etching were also seen (Figure 3b). With further etching, perikymata appeared more pronounced, and a typical honeycomb appearance was observed (Figures 4a, 4b). Examination of the enamel surface after debonding orthodontic brackets revealed covering the surface with a layer of bonding material, closing most of the preexisting pores in the first group, although the surface exhibited some irregularities, representing bulk remnants of the bonding material deposited in granular or globular patterns (Figure 3c).

After the de-bonding of the second group, some enamel rods with destructed cores were clearly seen, although the surface exhibited fewer bulging remnants than in the first group (Figure 4c).

## Discussion

The primary goal of this study was to assess the bonding strength of orthodontic brackets and tooth surfaces. When hypomineralized teeth were further etched, scanning revealed that perikymata became more pronounced and the typical honeycomb appearance was seen. However, after debonding, some enamel rods with destroyed cores were clearly visible, while the other group had a preserved enamel surface and exhibited fewer bulging remnants than the first group. Numerous studies have connected an improvement in the bond between the orthodontic bracket and the tooth to the effectiveness of the tooth's surface, its mineral content, the concentration of acid, and the length of the etching process. When Naji Kharouf and colleagues examined several etching techniques on healthy teeth and measured the shear bond strength, they discovered that rubbing in addition to etching strengthened the binding between this adhesive and the surfaces of the enamel [13]. 

Enamel is in continuous, dynamic communication with the mouth cavity ecosystem. The processes of demineralization and remineralization are always present, and their balance provides enamel integrity. If outer aggressive factors direct that balance towards demineralizing activities, the integrity of the crystal grid weakens, and the thickness and resistance of the enamel decline. Surpassing a certain border of enamel mechanic resistance leads to enamel fractures and cavity formations, as well as the beginning of irreversible damage [3]. Orthodontic treatment of children with hypomineralized enamel appears to be challenging. Due to the inferior adhesion to these teeth, there may be increased treatment times or even a poorer orthodontic outcome. These teeth could theoretically benefit from the application of preventive solutions, especially before bonding orthodontic brackets, which can promote further demineralization [9].

The bonding strength of orthodontic brackets to hypomineralized teeth after etching was greater than without etching, according to our findings. This finding is consistent with prior research, which found that the etch-and-rinse approach provides superior adherence to the two-component self-etching system, whether on fluorotic or healthy enamel [7,8]. Since the demineralization pattern in the second group appeared more regular than in the first group, greater bonding material diffusion in the enamel resulted in fewer surface remnants, as seen by group II's considerably smoother surface. The mild demineralization used to simulate enamel hypomineralization in the first group led to uneven penetration of the bonding material, where some of the bonding agents failed to penetrate the enamel and instead formed irregular deposits on the surface.

Teeth with mineral deficiency defects typically have significant adhesive failure rates because the surface features of the underlying enamel prohibit the etching patterns found in the enamel from obtaining results [14].

The current study's findings were also in agreement with another study that found that applying 37% phosphoric acid before self-etching results in greater gumminess rates than self-etching [10]. The bonding agent's penetration and diffusion into the demineralized enamel facial surface were critical for enamel adherence. The resin's capacity to permeate between the enamel crystallites and rods was largely responsible for the binding strength of the phosphoric acid-etched enamel. The high bond strength values reported with the ER [1,2], might have been due to this ultrastructure. This is a statistically significant difference. In the current investigation, scanning revealed an uneven surface with randomly dispersed tiny holes, suggesting a disturbed prism structure in some locations in hypomineralized dental enamel. Further etching with 37% phosphoric acid produced a characteristic honeycomb pattern owing to the preferred dissolution of the enamel prism cores, which might lead to higher adhesive material penetration and increased bond strength. After debonding, the acid-etched group's destructed cores could be seen more clearly through the enamel surface than the non-etched group. The second group's etching could be a viable method for providing enough adhesion in teeth with enamel imperfections, but there was a danger of greater surface loss and additional decalcification of enamel near orthodontic attachments.

This study's conclusion that brackets bonded to hypomineralized enamel without prior etching had a much lower SBS than the other groups was in line with the findings of other studies on hypomineralized teeth [1,2,5,15].

Because of the study's foreseen limitations, which included uncommonly collected molar incisor hypomineralized affected natural teeth, we adopted the present simulated hypomineralized approach. The outcomes of the present study indicate the need for further research with larger sample sizes to navigate treatment and orthodontic solutions with hypomineralized teeth.

## Conclusions

The results of the current study revealed that the adhesiveness levels were greater in teeth exposed to acid etching than in those bonded without etching. Bonding orthodontic brackets to hypomineralized enamel with no prior etching can be advantageous to better preserve the remaining enamel cores, but the shear bonding strength would suffer, as concluded above. It is advised to employ mechanical fixation techniques on hypomineralized affected teeth other than etching. A delicate balance is required to achieve adequate shear bond strength without compromising the remaining tooth structure.
